# Health system governance assessment in protracted crisis settings: Northwest Syria

**DOI:** 10.1186/s12961-023-01042-1

**Published:** 2023-08-30

**Authors:** Maher Alaref, Orwa Al-Abdulla, Zedoun Al Zoubi, Munzer Al Khalil, Abdulkarim Ekzayez

**Affiliations:** 1 Research for Health System Strengthening in Northern Syria (R4HSSS), Union for Medical and Relief Organizations, Incili Pinar MAH, 27090 Gaziantep, Turkey; 2 Strategic Research Center (ÖZ SRC), Gaziantep, Turkey; 3 Syria Public Health Network, London, UK; 4 The Centre for Conflict & Health Research (CCHR), King’s College London, London, UK

**Keywords:** Central bodies, Conflict, Crisis, Governance, Health system, Syria

## Abstract

**Background:**

Since the withdrawal of government forces from Northwest Syria due to the conflict, several national initiatives have aimed to create alternative governance approaches to replace the central governmental system. One of the recent initiatives was the formulation of so-called ‘Central Bodies’ as institutional governance structures responsible for thematic planning and service provision; for example, the referral unit is responsible for planning and delivering medical referral services. However, the governance and administrative rules of procedures of these bodies could be immature or unsystematic. Assessing the governance of this approach cannot be condoned, especially with the urgent need for a methodical approach to strategic planning, achieving strategic humanitarian objectives, and efficiently utilizing available resources. Multiple governance assessment frameworks have been developed. However, none were created to be applied in protracted humanitarian settings. This research aims to assess the extent to which the existing health governance structure (central bodies) was capable of performing the governance functions in the absence of a legitimate government in Northwest Syria.

**Methods and materials:**

A governance assessment framework was adopted after an extensive literature review and group discussions. Four principles for the governance assessment framework were identified; legitimacy, accountability and transparency, effectiveness and efficiency, and strategic vision. Focus Group Discussions were held to assess the levels of the selected principles on the governance thermometer scale. Qualitative and quantitative data were analyzed using NVivo 12 and SPSS 22 software programs, respectively.

**Results:**

The level of the four principles on the governance thermometer scale was between the lowest and middle quintiles; ‘very poor or inactive’ and ‘fair and requires improvement’, respectively. The results indicate that the governance approach of Central Bodies in NWS is underdeveloped and summons comprehensive systematic development. The poor internal mechanisms, poor planning and coordination, and the absence of strategic vision were among the most frequent challenges to developing the approach.

**Conclusion:**

Humanitarian actors and donors should pay more attention to health governance approaches and tools in protracted crises. The central bodies must improve coordination with the stakeholders and, most importantly, strategic planning. Establishing or utilizing an independent planning committee, with financial and administrative independence, is crucial to maintain and improving contextual governance mechanisms in Northwest Syria.

## Background

The Government of Syria GoS has relinquished many areas in Syria as a result of the war against the various military factions since 2011 [1]. Some of these areas, such as Northwest Syria NWS, were left without a systematic governance system, and the response to the population needs in these areas has become dependent on meeting the demands at the level of communities or sub-districts [2, 3]. Many humanitarian Non-Governmental Organizations NGOs initiated response operations in NWS under the umbrella of the United Nations UN humanitarian coordination system by assessing people's needs at the level of individuals and communities without clear governmental structuring objectives due to the severity of the emergency, especially with the widespread collapse of the infrastructure, the poor socioeconomic situation and the continuous attacks on healthcare centers [4, 5].

The health system governance in Syria is collapsed and fragmented due to the multiple power of control in the region and the absence of a central national system [6, 7]. Since the withdrawal of government forces from NWS, several national initiatives have aimed to create governing bodies to compensate for the absence of the government health system. One of these attempts was the formation of Idleb Governorate Medical Commission in 2012, an immediate predecessor of the provincial Health Directorates (HDs), which was established in 2013 through the initiatives of local medical cadres [8, 9]. Later in 2013, the Interim Syrian Ministry of Health MoH formally established the HDs in several governorates in NWS [10]. While the supposed role of these directorates as local authorities was to rebuild the collapsed health system, their roles were relegated mainly to coordination and service delivery instead of system recovery and administration. This shift in roles was expected due to the lack of specialized expertise, shortage of resources, and attacks on health services [11].

Establishing a quasi-state health governance structure in NWS encouraged donors to support the HDs [12]. Many international donors supported the governance initiative in NWS through NGOs. However, International donors suspended support to the HDs following the military advance of Hay'at Tahrir al-Sham HTS (formerly an al-Qaeda affiliate known as Jabhat al-Nusra) over Idleb and parts of Aleppo Governorates in 2019 due to counter-terrorism measures [13, 14]. Donors started to shift their funds from the local HDs to other organizations, which resulted in weakness in the administrative capacity and roles of the directorates [7], and prompted health directorates, local doctors, and many Syrian organizations to support alternative governance systems through the formation of so-called central bodies. The most prominent bodies were the Syrian Board of Medical Specialties (SBOMS), Syria Immunization Group (SIG), Infection Prevention and Control (IPC body), Referral Network, and Health Information System (HIS) unit (or District Health Information Unit DHIS) [4, 7, 15–17].

Financial support has come from multiple donors. For example, World Health Organization (WHO) supported an integrated approach to service delivery in NWS by funding the referral network of Harim, Idleb (mobile and fixed health services, hospitals, and referral vehicles) [18]. DHIS was established in NWS to monitor the progress against the health and nutrition cluster indicators. While the DHIS was operated by the central body of HIS, health partners were responsible for feeding the system with the required data. This system was founded and supported by WHO to replace the collapsed information management system in areas out of Government control [19]. An assessment conducted in NWS between 2019 and 2020 revealed high levels of viral infection within the health facilities due to the lack of IPC measures [20]. For that, WHO supported IPC program in NWS through technical capacity building, provision of personal protective equipment, and financial resources [21]. In addition, WHO, in cooperation with other donors and humanitarian actors, supported the service delivery in NWS by developing the Essential Health Service Package guidelines in 2016 and establishing networks of referral services with standard procedures and service provision protocols [18]. Due to the Syrian governmental absence in NWS, these bodies were established in the presence of the de-facto health authorities in the region [7].

The bottom-up development of the current governance system of central units in NWS resulted from the necessity to respond to the health needs of affected communities [15]. Relevantly, this approach is identified as an institutional governance system when compared to the three approaches of health system governance [22] because it was shaped based on the community's demand for health services [23]. The institutional governance approach follows organizational and individual interests and initiatives to respond to timely needs instead of long-term national goals by carrying out identified tasks within specific themes in the health sector [24]. This definition corresponds to some extent with the current health governance approach of the central bodies in NWS, which were formed to implement specific health services within a particular scope. For example, the Syrian Immunization Group is responsible for coordinating the vaccination campaigns in NWS to increase access to immunization services in cooperation with WHO, United Nations Children's Fund UNICEF, and NGOs [16, 25]. The experience of central bodies is not the first worldwide. There have been some examples of central projects approach to implement thematic health interventions. Key features of such central projects include independence, autonomous structure, and technical focus. For example, some health system programs, like HIS, were framed institutionally within the vertical system of the Syrian MoH before the crisis [26]. In 2001, the fight against the Big Three (Human Immunodeficiency Virus HIV, Tuberculosis, and Malaria) was a practical test of navigating through political and economic complexity in fragile and conflict-affected African countries [27]. However, projects with central technical structures have some gaps and shortages in relation to the design and functionality of these structures that might result in poor implementation and poor health outcomes [28]. Several studies from conflict settings, like in NWS, found that much attention is usually paid to life-saving health interventions at the expense of health governance [29–31]. The assumption in such settings is that conflict disrupts local health systems with different levels of collapse in health leadership [32]. This might push humanitarian actors to establish a parallel health governance system to coordinate health response [33]. Like the abovementioned examples, one way to navigate these systems could be by establishing central technical structures or public health systems [34, 35].

## Health system governance

According to Brinkerhoff and Bossert, governance in health systems is the process of developing and implementing effective institutional rules for policies, programs, and activities related to fulfilling public health functions in order to achieve health sector objectives [36]. Abimbola identified three approaches to conceptualizing health system governance based on the hardware-software framework of what constitutes a health system; the government-centered approach, which focuses on governments roles in the governance system, regardless of non-government health system actors; the building-block approach, which focuses on the internal workings of decentralized government ministries and health facilities; and the institutional approach which focuses on health system software [37]. Hardware refers to the concrete and tangible conceptions of health systems management, such as general budget, pharmaceuticals, information management, human capital, infrastructure, and the organizational structures to provide policies, services, and interventions, as well as their intended targets, users, and beneficiaries. Software refers to the quantifiable components of health systems, such as the goals and interests, values and norms, relations between the different actors, and power [38]. These definitions confirm the aforementioned conclusion that the current health governance in NWS fits the institutional system, where software, like the interests of NGOs and donors in responding to the community health demands, power and authorities of existing stakeholders, and the goal to maintain health service delivery overshadow the system management and development.

## Health system governance assessment

A systematic assessment of governance, as one of the health system's building blocks, is essential to influencing the system's performance [39]. Although several frameworks for health governance were developed [40], all of these frameworks were created for stable settings with very limited applicability in armed conflict settings [41]. Governance is included in the concept of stewardship, which WHO defined as "the careful and responsible management of the population's well-being" [42]. While several frameworks for assessing health system governance have been developed, their application is hampered by unapplicable indicators or is overly complex [43].

Islam (2007) argued that health governance is based on the World Governance Indicators (WGI) and health determinants [44]. (WGI) was developed by the World Bank to rate the country’s health system based on six indicators: accountability, political stability, effectiveness, rule of law, regulatory quality, and control of corruption [45]. In their policy brief to the U.S. Agency for International Development USAID (2008), Brinkerhoff and Bossert underlined the concept of good governance as a goal that is achieved when all the actors in the health sector fulfill the governance principles, including accountability, transparency or open policy process, leadership, legitimacy, and efficiency [36]. Karimi and Shafaee investigated the health system governance in Syria based on eight characteristics described by United Nations Economic and Social Commission for Asia and the Pacific [46]; participation, rule of law, transparency, responsiveness and accountability, consensus-oriented, equity and inclusiveness, effectiveness and efficiency, and accountability. The authors found that the current unstable political conditions, dictatorship, and one-family reign contributed to a deteriorated governance system in Syria [47]. Siddiqi et al. presented an analytical framework of 10 principles to asses the health system governance at the country level. These principles are; strategic vision, participation, rule of law, transparency, responsiveness, equity, effectiveness and efficiency, accountability, information, and ethics. The authors defined each of these principles and divided the assessment into three levels; policy implementation, national, and health policy formulation. Mikkelsen-Lopez et al. developed an approach to addressing governance from a health system framework perspective. The governance elements of this approach are participation, strategic vision, addressing corruption, transparency, and accountability [43]. Global and UN agencies, like World Bank, WHO, Pan American Health Organization PAHO, and United Nations Development Program UNDP, have developed different models to assess health system governance. The WHO framework identified several domains based on the stewardship concept, including information management, policy formulation, implementation tools, partnership, and accountability [48]. PAHO governance assessment framework comprises 11 principles related to Essential Public Health Functions EPHF, including quality assurance, research, policy development, and surveillance [49, 50]. World Bank framework identified six fundamental aspects of governance; accountability, political stability, effectiveness, regulatory burden, rule of law, and control of corruption [51]. UNDP assessment framework proceeds from identified thematic areas of the health system; meaningful participation and legitimacy, responsiveness and performance, strategic vision and direction, accountability and efficiency, and equity and rule of law [52]. Pyone et al., in a systematic review of the health system governance assessment, identified 16 frameworks categorized based on development theory into four disciplines; institutional economics, political science and public administration; international development; and multidisciplinary. Notwithstanding, none of them is applicable in protracted crisis settings [40].

The development of health systems in contexts of protracted conflict is usually unsystematic, lopsided, and of an arbitrary nature, determined by certain factors that vary according to the context [53]. In addition, uncertainties in the prolonged political crisis, like in NWS, affect the governance model in different sectors [54]. This paper, which could be the first in a protracted humanitarian setting, aims to assess the extent to which the existing health governance structure was capable of performing the governance functions in the absence of a legitimate government in NWS based on a hybrid health governance assessment framework adopted for the context.

## Methods and materials

This study aims to assess the health system governance in NWS, which has been impacted by a violent war, outbreaks, collapsed infrastructure, and a lack of resources for over a decade [55]. In line with the research framework, westarted with a brief history of the crisis and its impact on the health system and health governance in Syria, and the initiatives to rebuild an alternative health system in non-governmental areas, particularly NWS, including the formation of HDs and central bodies modality. Later, the research touched upon available literature on governance and governance assessment frameworks. Throughout the research framework development, it was challenging to identify health governance assessment frameworks in protracted emergency settings to be utilized in the context of NWS. Therefore, an adapted framework with assessment principles applicable to the context was deducted through a two-day workshop in November 2021. The participants in the workshop were stakeholders, leaders, and decision-makers from the humanitarian and academic organizations in Turkey, as these organizations are part of the coordination platform of the humanitarian intervention in NWS, which is based in Turkey [56]. The participants were selected based on the following criteria: leadership roles in the health sector in NWS and relevant background to the research topic. An invitation email was sent by the research project team to the selected participants. Assistance recipients were not involved in the discussions for many reasons, including access to Turkey from NWS, safety and security issues, and the technical nature of the research topic might not be known by people outside the health sector.

One of the workshop's objectives was to extract governance assessment principles based on the participant's understanding of the research concept; health system governance assessment. Therefore, contextualized scientific knowledge of the research concepts was developed on the first day through interactive sessions on the previous and current health governance initiatives and available governance assessment mechanisms in NWS. In consonance with the participants' feedback and inputs, four thematic principles were deduced to assess the health system governance; legitimacy, transparency and accountability, effectiveness and efficiency, and strategic vision. Later, the research team identified and adapted relevant and context-specific definitions and indicators for these principles in accordance with the reviewed literature. A scale of 1 to 10 was suggested to measure the functionality of the four principles, which was converted to a percentage and reflected in a governance thermometer scale of 0% (very poor) to 100% (excellent) [57]. For this research, the scale was adapted by adding interscale ranges; (1) 0–20%: very poor or inactive; (2) 21–40%: poor and requires significant improvement; (3) 41–60% fair and requires improvement; (4) 61–80% good; and (5) 81–100% perfect. Four central projects have been selected for the health governance assessment; HIS, IPC, referral, and SBOMS units.

On the second day, the definitions of the four selected principles were presented to the attendees for in-depth discussion from a scientific perspective. Four Focus Group Discussions FGDs, one for each of the selected central bodies, were conducted to assess the health system governance of the central bodies approach in NWS based on the four principles and relevant definitions and indicators. The invitees who accepted to join the FGDs had to sign a consent form. All the consent forms were stored in the King’s College archive and accessible only by the principal researcher. The participants were divided into the FGDs based on their relevant experience, area of work, and educational background. The participants were asked to provide a quantitative measurement of the level of functionality of the health system governance against each of the four principles for the selected central bodies based on the governance thermometer scale. Semi-structured questions were administered later to allow the participants to present qualitative justification for the given level of functionality.

Data were anonymized and transcribed in Arabic, which was translated into English later by an official translator. Qualitative and quantitative data were analyzed using NVivo 12 software and SPSS statistics software v22, respectively.

Research framework, transcripts, definitions, questionnaires, written consent form, ethical clearance, and data analysis extractions are available at https://doi.org/10.7910/DVN/K0A252.

## Results

### Sample characteristics

A total of 24 individuals from multiple entities participated in the discussion. The participants were from NGOs, donors, SIG MoH and HDs, and central bodies. Each FGD was divided into four main sessions relevant to the adapted health governance assessment principles. While all the participants have Syrian nationality, most live either in Turkey or Europe. More than 80% of the participants were physicians in senior positions (manager or coordinator). It is worth mentioning that all the participants were males, although the invitation to the workshop was circulated to a broad spectrum of NGOs, local authorities, and UN agencies, which might reflect gender imbalance in the health sector in NWS.

### Health governance legitimacy

Health system governance legitimacy was defined as the belief that a rule, institution, or leader has the right to govern. It is an individual judgment about the rightfulness of a hierarchy between the ruler and the subordinate’s obligations toward the rule or ruler [58]. In the case of NWS, the ruler is the head of the central bodies, and the subordinate is the NGOs that should cooperate with the central body to achieve particular thematic public health objectives.

The majority of the answers about the legitimacy of the central bodies approach were on the 3rd and 4th quintiles on the thermometer scale (Table 1). While 37% of the participants said that the legitimacy level of the current governance approach requires improvement, 46% said that the legitimacy level is good. The mean legitimacy level on the governance thermometer scale was 57%.

**Table 1:** NWS Health system governance assessment—legitimacy level according to the governance thermometer scale

Thermometer scale	Frequency	Percent	Valid percent	Cumulative percent
*Valid*				
No answer	2	8.3	8.3	8.3
fair and requires improvement	9	37.5	37.5	45.8
good	11	45.8	45.8	91.7
very poor or inactive	2	8.3	8.3	100.0
Total	24	100.0	100.0	

When the participants were asked about the challenges to improving legitimacy (multiple answers per participant), the most frequent challenge (23%) was ‘competition and the lack of unification across the health sector’. Competition refers to the negative contest between NGOs for financial support. Other frequent challenges were ‘the weak cooperation by NGOs with the central bodies’ and ‘weakness in the internal system or operational model and project design’.

“There are three sources of legitimacy: NGOs, WHO, and the health cluster, besides the willingness and possibility to support HIS unit, and donors who have the power to impose cooperation with the HIS on their implementing partners.”

Table 2 shows the challenges to improving the legitimacy of the health system in NWS according to the participant's views.

**Table 2:** NWS health system governance—legitimacy challenges

	Responses		Percent of cases
	*N*	Percent	
*NWS health system governance legitimacy challenges*			
Competition and the lack of unification across the health sector	14	23.0	58.3
The fragmented governance structure	8	13.1	33.3
The lack of resources and poor sustainability planning	7	11.5	29.2
The political aspect of the crisis	2	3.3	8.3
The poor support from WHO and the health cluster	5	8.2	20.8
The weak cooperation by NGOs	10	16.4	41.7
Underestimating the importance of the central bodies approach	6	9.8	25.0
Weakness in the internal system or operational model and project design	9	14.8	37.5
Total	61	100.0	254.2

**Fig. 1:**
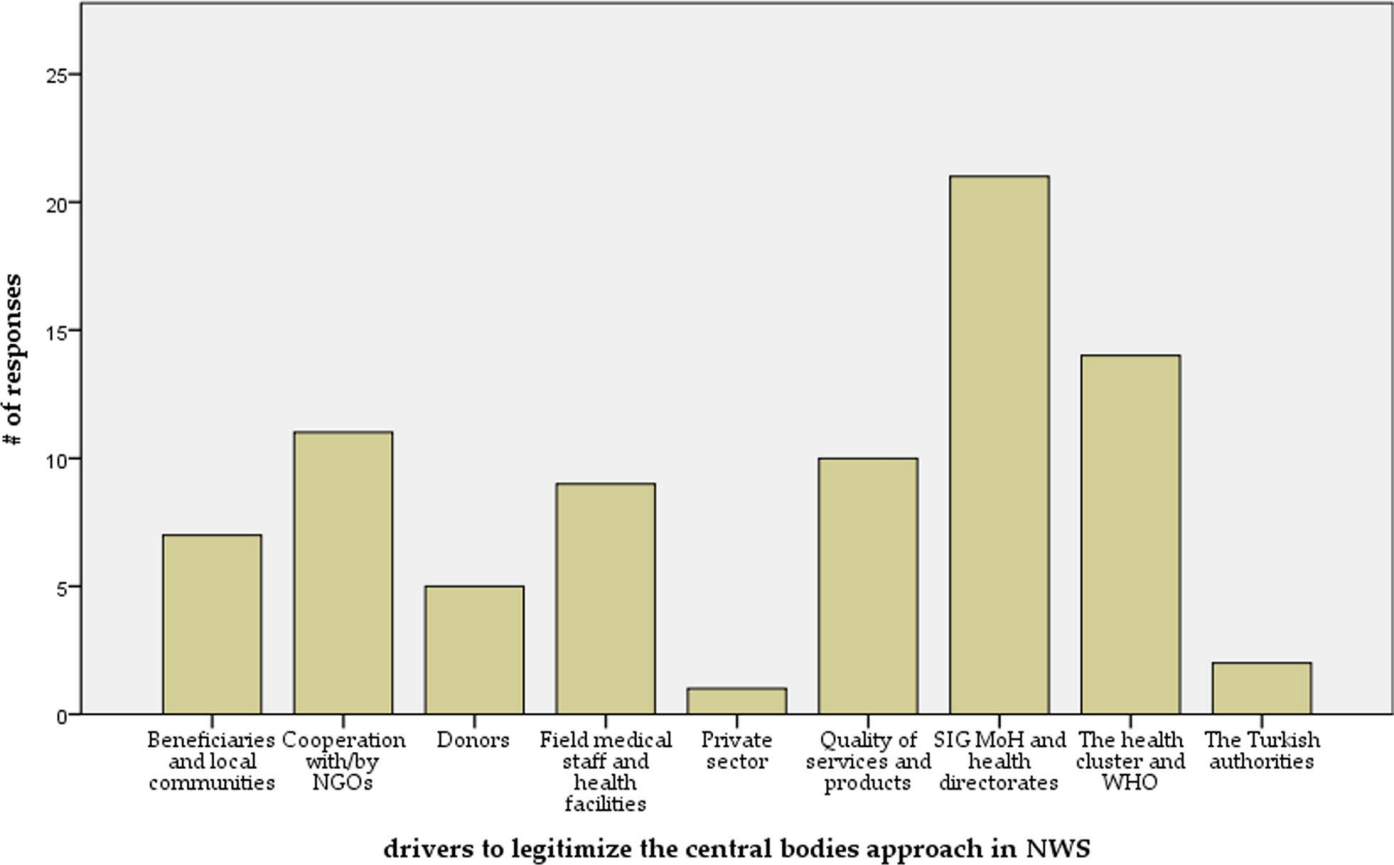
NWS health system governance—drivers to legitimize the central bodies approach in NWS

When the participants were asked about the institutional factors that drive more legitimacy to the central bodies' governance approach, it was found that cooperation with SIG MoH and its HDs, WHO and the health cluster, and NGOs was the most important driver to legitimize the current health governance approach in NWS. Quality of services and products was one of the most frequent factors contributing to legitimizing the central bodies, according to the participants. Figure 1 shows the drivers to legitimize the health system governance in NWS.

### Health governance accountability and transparency

Transparency and accountability can reduce the risk of corruption and unethical behavior while increasing public trust in government institutions [59], [60]. Transparency and accountability are interlinking elements in governance systems [61]. Transparency refers to the public availability of usable information, which mitigates corruption risks and allows scrutiny of rulers' decisions [62]. The heads of the central bodies (rulers) explained the existing transparency and accountability mechanisms to the attendees and presented the hierarchy of each of the bodies. The facilitators, thereafter, raised questions on the current level of accountability and transparency of the central bodies and investigated the challenges and factors to enhance accountability and transparency mechanisms.

Almost 66% of the answers were on the 1st and 2nd quintiles of the governance thermometer scale, referring to an inactive or poor accountability mechanism within the central bodies (mean *μ* ≈ 30%) (Table 3).

**Table 3:** NWS Health system governance assessment—accountability and transparency level according to the governance thermometer scale

Thermometer scale	Frequency	Percent	Valid percent	Cumulative percent
*Valid*				
No answer	2	8.3	8.3	8.3
fair and requires more improvement	5	20.8	20.8	29.2
good	1	4.2	4.2	33.3
poor and requires significant improvement	5	20.8	20.8	54.2
very poor or inactive	11	45.8	45.8	100.0
Total	24	100.0	100.0	

The answers of the participants were based on their practical experience in coordination and work with central bodies. Most of the participants debated how the weak accountability and transparency mechanism has caused challenges associated with administrative and field coordination between the NGOs and central bodies.

“There is a lack of clarity in the administrative structure and the decision-making mechanism, which created challenges in the governance of the central system, which is supposed to support the health sector governance.”

Along with the inactive or poor accountable governance system in the health sector, the participants mentioned various challenges preventing the health sector in NWS from building a rigorous accountability and transparency mechanism. Among these challenges (Table 4), ‘weakness in the internal system or operational model and project design’ was the most mentioned by the participants (87%), followed by ‘the lack of resources and poor sustainability planning’ and ‘the lack of legitimacy’.

“The low turnover rate of the position for a long time is one of the central bodies weaknesses.”

“The head of the central body is accountable to the provincial health directors. However, none of them can terminate his contract or hold him accountable.”

**Table 4:** NWS health system governance—accountability and transparency challenges

	Responses		Percent of cases
	*N*	Percent	
*NWS health system governance accountability and transparency challenges*			
Competition and the lack of unification across the health sector	2	3.8	8.3
The lack of independence, neutrality, and objectivity	3	5.8	12.5
The lack of legitimacy	8	15.4	33.3
The lack of resources and poor sustainability planning	9	17.3	37.5
The poor support from WHO and the health cluster	1	1.9	4.2
The weak cooperation by NGOs	4	7.7	16.7
The weak capacity	4	7.7	16.7
Weakness in the internal system or operational model and project design	21	40.4	87.5
Total	52	100.0	216.7

### Health governance effectiveness and efficiency

**Fig. 2:**
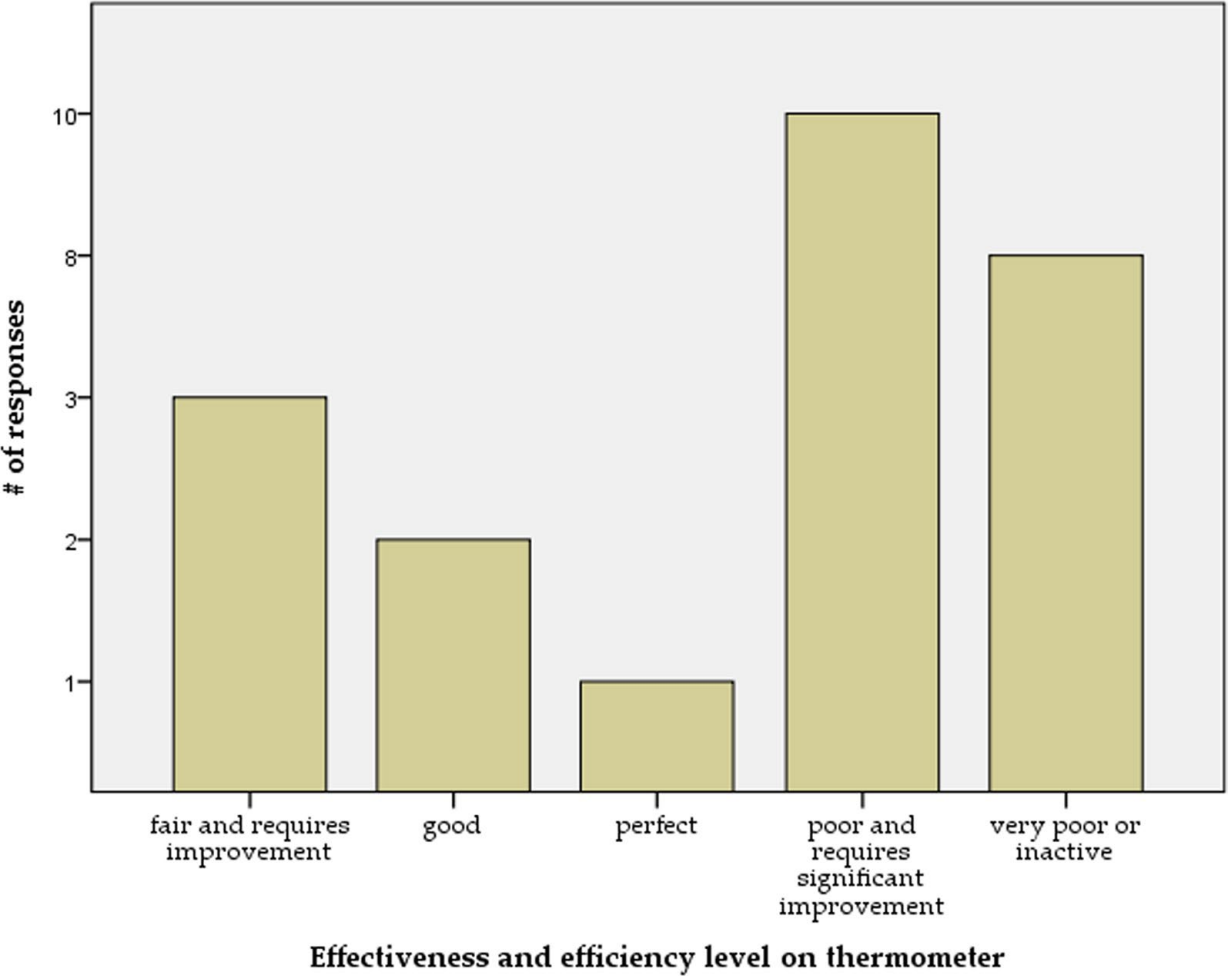
NWS Health system governance assessment—effectiveness and efficiency level

Effective and efficient governance is critical to the well-being of any country [63]. Effectiveness refers to fulfilling the required task rightfully, and efficiency is fulfilling these tasks in the most economical way in terms of resources and time [64]. The facilitator presented these definitions with practical and actual examples to the attendees in an interactive session. Later, questions on the level of effectiveness and efficiency of the health system governance were raised to the participants. The majority of the participants indicated a poor or a very poor level of effectiveness and efficiency of the central bodies approach (Fig. 2). The mean level of effectiveness and efficiency on the governance system thermometer scale was 33%, referring to the central bodies' poor effectiveness and efficiency mechanism.

“By comparing the inputs to the outputs and observing whether the best methods were used to reach the goal, I can say that perhaps the best efforts were made but not the best performance. Knowing what resources were made available to the central bodies and understanding the primary goal is necessary.”

In fact, 75% of the participants pointed out that the current mechanism is either inactive or requires significant improvement.

The challenges to enhancing the system's effectiveness and efficiency (Table 5) were mainly related to weaknesses in the internal system or operational model and project design and the lack of resources and sustainability planning (50% and 42% of the participants, respectively).

**Table 5:** NWS health system governance—effectiveness and efficiency challenges

	Responses		Percent of cases
	*N*	Percent	
*NWS health system governance effectiveness and efficiency challenges*			
Competition and the lack of unification across the health sector	1	3.1	4.2
cooperation with/by NGOs	3	9.4	12.5
The lack of legitimacy	3	9.4	12.5
The lack of resources and poor sustainability planning	10	31.3	41.7
The poor support from WHO and the health cluster	1	3.1	4.2
Underestimating the importance of the central bodies approach	2	6.3	8.3
Weakness in the internal system or operational model and project design	12	37.5	50.0
Total	32	100.0	133.3

### Health governance strategic vision

According to [65], strategic vision refers to having a broad and long-term perspective on health planning and development and a sense of strategic direction for such development. Based on this definition, and according to the FGDs outputs, non of the four central projects has a clear strategic vision or a documented rigorous strategic plan. Strategic planning is embedded in the program design. However, there is no systematic process for long-term contextualized strategic planning. Moreover, at the level of health stewardship, there was no clear sense of development and dealing with the determinants of health and the political complexities associated with the health sector.

“Health governance is absent in SBOMS. There is no future vision of the goals, strategic planning, and accountability system.”

“The central bodies must develop a strategic vision of the health system in Syria.”

When participants were asked if there was a clear strategic plan and engagement with organizations and stakeholders (Table 6), 96% of the answers were within the lowest quintile of the governance thermometer scale, and the mean level was *μ* ≈ 12%.

**Table 6:** NWS Health system governance assessment—strategic vision level according to the governance thermometer scale

Thermometer scale	Frequency	Percent	Valid percent	Cumulative percent
*Valid*				
Fair and requires improvement	1	4.2	4.2	4.2
Poor and requires significant improvement	2	8.3	8.3	12.5
Very poor or inactive	21	87.5	87.5	100.0
Total	24	100.0	100.0	

**Fig. 3:**
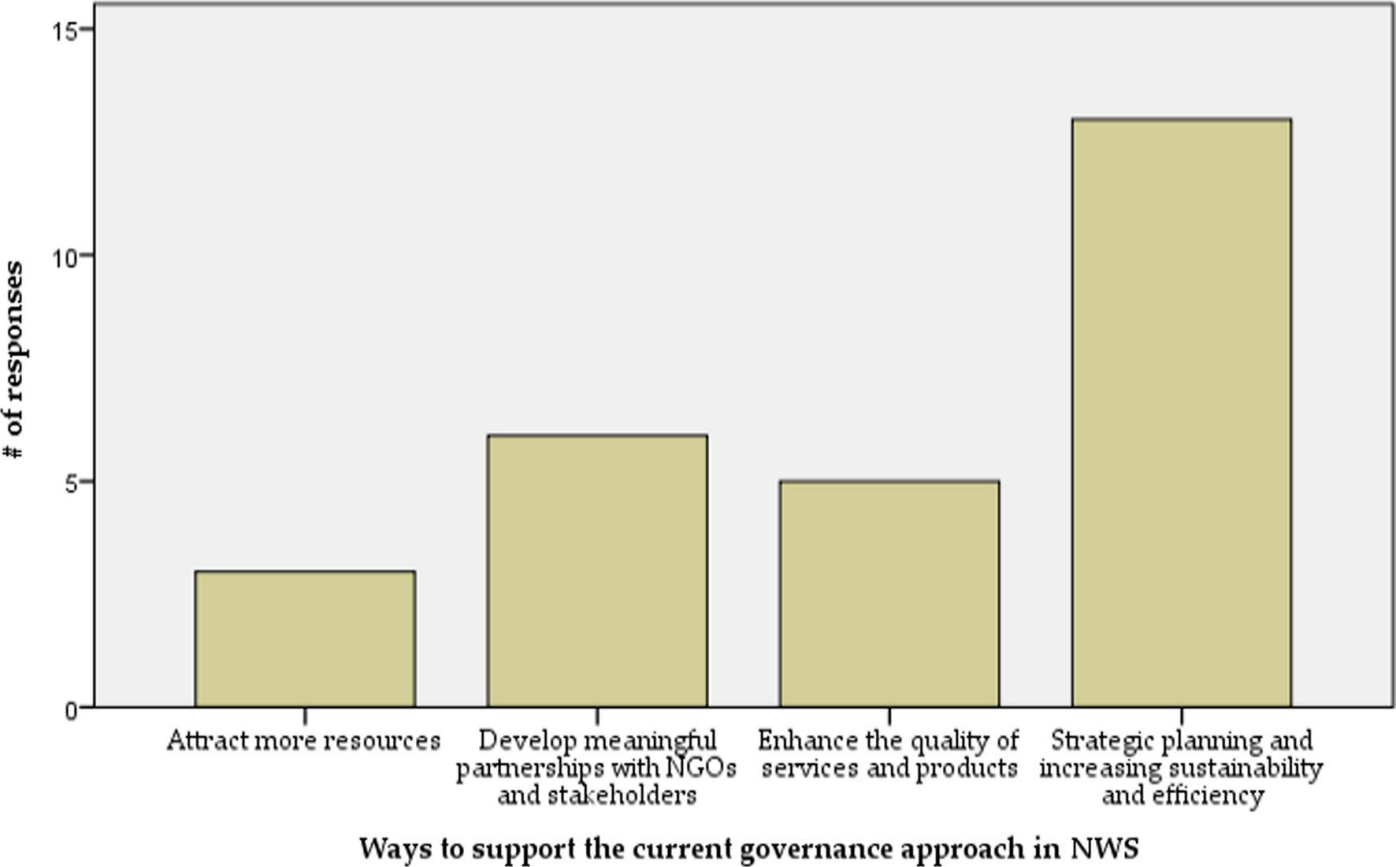
NWS Health system governance assessment—ways to strategically support the central bodies' approach

Based on the participants' views, ‘strategic planning and increasing sustainability and efficiency’ is the most crucial way to support the central bodies' approach in NWS for sustainable services, especially during the health system recovery phase (Fig. 3).

Other ways to support the current governance approach in NWS were ‘develop meaningful partnerships with NGOs and stakeholders’, ‘enhance the quality of services and products’, and ‘attract more resources’.

“The central units should be more efficient in investing resources, especially in light of the lack of resources. Thus, improving efficiency is key to marketing this approach and convincing donors to support the central projects. Additionally, service quality and performance should be enhanced.”

## Discussion

The research aims to explore and assess the existing health system governance in NWS by applying an adapted framework assessment after an extensive literature review of the relevant literature. In fact, all of the reviewed framework assessments were limited to stable states and systems. Therefore, an assessment framework was deduced based on the research team's contextual understanding of the current NWS governance system within the scope of the reviewed literature through a two-day workshop in Turkey. During this workshop, participants from various institutions presented detailed explanations of the historical brief of the current health sector governance system. The workshop also reviewed the current experiences of the so-called central bodies and their governance mechanisms. Group sessions with participants followed these activities to assess the governance of central bodies based on the deducted assessment framework. The governance assessment framework principles were legitimacy, transparency and accountability, effectiveness and efficiency, and strategic vision.

**Fig. 4:**
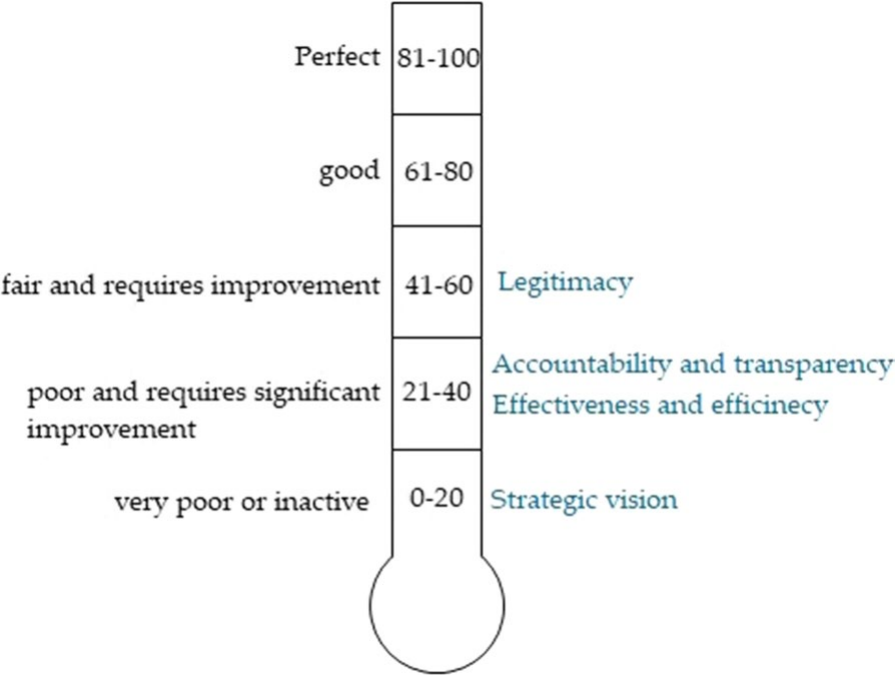
Health system governance thermometer scale: the position of the four principles of health governance assessment on the governance thermometer scale—NWS. Adapted from [57]

The research assessed the level of each of the four principles on the health governance thermometer scale (Fig. 4). The legitimacy of the institutional approach of the central bodies in NWS is fair and requires improvement by increasing coordination with SIG MoH and its HDs, NGOs, and WHO and the health cluster. While field health facilities, communities and beneficiaries, and quality of services and products were remarkable drivers to legitimize the current health system governance, donors as one of the legitimacy factors were not a significant reason to increase the health governance legitimacy. In addition, NGOs must develop sectoral coordination to contribute to the strategic objectives of the central bodies which have, in turn, to improve cooperation with NGOs and other stakeholders to achieve a perfect legitimacy level on the governance thermometer scale.

According to the central bodies' management, the accountability and transparency mechanism is immature and not appropriately documented. This is confirmed by the fact that the turnover rates in the central projects' managerial positions are almost non-existent. Nonetheless, activating accountability mechanisms is not an independent procedure. It entails changing dynamics, assigning roles and responsibilities, engaging communities meaningfully, and developing an inclusive strategic vision [66–68]. Moreover, almost a third of the participants mentioned that the lack of legitimacy is one of the reasons that hinder the development of the accountability system, which is what was mentioned by Brinkerhoff that accountability and legitimacy are interlinked concepts of governance in the eyes of stakeholders [69]. The weakness in the internal system or operational model is one of the most mentioned challenges to enhancing the principles levels on the governance thermometer scale. Dull internal systems, poor management and leadership, and lack of resources are interlinked with poor strategic planning [70], meaning that developing contextualized and practical strategies is an inevitable factor in improving the governance system of the central bodies. Improving strategic vision in the health sector might entail creating an autonomous statutory planning committee, separate from the health authorities, with sufficient financial leverage [71]. In the context of NWS, the planning committee could be from the health cluster members or an established body by the NGOs. The health cluster is accountable to WHO and responsible for planning and coordinating humanitarian intervention in NWS with access to sources of funds through advocacy and communication functions [72]. Therefore, developing an integrated and comprehensive strategic plan in which all stakeholders contribute, based on the objectives sought by central bodies and organizations, is a cornerstone for improving the health sector governance system in NWS.

## Conclusion

Central projects represent an innovative approach to health governance in chronic conflict settings where health governance is generally neglected. Delaying governance support during emergencies could be of disastrous impact on service delivery and quality control. Humanitarian actors and donors should pay more attention to health governance approaches and tools in protracted crises to enhance resource utilization effectiveness and efficiency. Besides improving the coordination with all stakeholders, accountability mechanisms, and efficiency, central bodies must concentrate on strategic vision and planning. Due to the lack of resources, shifting to strategic planning might be at the expense of service delivery. Therefore, formulating a committee for this purpose with financial and administrative independence is crucial at this stage for strategic planning and comprehensive improvement in the health system governance in NWS. This research was silent on the health economics aspects of the governmental structure in NWS. Therefore, pursuing further research to investigate and study the cost efficiency of this approach is recommended to complement and support advocacy for health governance in conflict settings.

## Data Availability

The datasets generated and/or analyzed during the current study are available in the Harvard Dataverse repository, https://doi.org/10.7910/DVN/K0A252

## Abbreviations

DHIS: District Health Information System
EPHF: Essential Public Health Functions
FGDs: Focus Group Discussions
GoS: Government of Syria
HD: Health Directorate
HIS: Health Information System
HIV: Human Immunodeficiency Virus
HTS: Hay’at Tahrir al-Sham
IPC: Infection Prevention and Control
MoH: Ministry of Health
NGO: Non-Governmental Organization
NWS: Northwest Syria
PAHO: Pan American Health Organization
SBOMS: Syrian Board Of Medical Specialities
SIG: Syrian Interim Government
SPSS: Statistical Package for Social Science
UN: United Nations
UNDP: United Nations Development Program
UNICEF: United Nations Children’s Fund
USAID: U.S. Agency for International Development
WGI: World Governance Indicators
WHO: World Health Organization
